# Targeting the Calcium-Sensing Receptor in Chemically Induced Medium-Grade Colitis in Female BALB/C Mice

**DOI:** 10.3390/nu16244362

**Published:** 2024-12-18

**Authors:** Karina Piatek, Valeriya Gushchina, Ava Kleinwächter, Nadja Kupper, Ildiko Mesteri, Taha Elajnaf, Luca Iamartino, Martina Salzmann, Christian Müller, Teresa Manhardt, Andrea Vlasaty, Enikö Kallay, Martin Schepelmann

**Affiliations:** 1 Institute for Pathophysiology and Allergy Research, Medical University of Vienna, Waehringer Guertel 18-20, 1090 Vienna, Austria; karina.piatek@meduniwien.ac.at (K.P.); enikoe.kallay@meduniwien.ac.at (E.K.); 2 Pathologie Überlingen, 88662 Überlingen, Germany; 3 Nuffield Department of Women’s and Reproductive Health, Medical Sciences Division, University of Oxford, Oxford OX3 9DU, UK

**Keywords:** calcium-sensing receptor, calcium, inflammatory bowel disease, colitis, NPS 2143

## Abstract

Background/Objectives: The extracellular calcium-sensing receptor (CaSR) is a multifunctional receptor proposed as a possible drug target for inflammatory bowel disease. We showed previously that CaSR inhibition with NPS 2143, a negative allosteric modulator of the CaSR, somewhat ameliorated the symptoms of chemically induced severe colitis in mice. However, it was unclear whether the potential of CaSR inhibition to reduce colitis may have been overshadowed by the severity of the induced inflammation in our previous study. Therefore, we tested if CaSR inhibition could prevent medium-grade colitis. Methods: Female BALB/c mice were treated with NPS 2143 or a vehicle prior to the induction of colitis with 2.5% DSS. On the day of sacrifice, colons and plasma were collected. The histology score was determined based on hematoxylin-eosin-stained sections. Mucin content, proliferation (Ki67), and immune cell infiltration (CD3 and CD20) were quantified based on immunostainings. Gene expression was measured by RT-qPCR. Results: Treatment with NPS 2143 had no effect on the clinical symptom score of the mice. However, the colons of the mice in the treated group were significantly longer (*p* < 0.05), and NPS 2143 significantly reduced colon ulceration (*p* < 0.05). The treatment also significantly reduced the expression of COX2 in the proximal colon and IL-22 in the distal colon. The proliferation of cells in the lymph nodes was significantly lower after the treatment, but no difference was observed in the epithelial cells. Conclusions: In summary, while NPS 2143 had an anti-inflammatory effect on medium-grade colitis, this effect appeared to be milder than in severe colitis, as observed previously, indicating that the effectiveness of CaSR inhibition as an anti-inflammatory measure in the colon is proportional to disease severity.

## 1. Introduction

Inflammatory bowel disease (IBD) is a set of chronic inflammatory disorders of the gastrointestinal tract that encompasses ulcerative colitis and Crohn’s disease. It is characterized by severe inflammation that leads to mucosal ulceration and epithelium remodeling, ultimately causing disruption to the epithelial barrier [1]. Although several factors in the pathogenesis of IBD have been identified, its exact etiology remains unknown [2]. Currently, there is no cure for IBD, and the treatment focuses mainly on symptom management, which is often accompanied by side effects [3].

The extracellular calcium-sensing receptor (CaSR) is a G protein-coupled receptor best known for its role in calcium homeostasis [4]. Over the past years, several other physiological roles of this multifunctional receptor have been discovered [5], among them the modulation of inflammation. Numerous studies have demonstrated a pro-inflammatory role of the CaSR in the lungs, adipose tissue, cardiovascular system, and brain [6,7,8,9,10]. The results for the colon are more heterogenous, the CaSR seems to be involved in maintaining epithelial integrity and fluid secretion [11] and is believed to have mainly anti-inflammatory effect [12]. Recent studies from our group, however, showed that, like in the other mentioned tissues, the CaSR has actually a pro-inflammatory role in intestinal cells, both in vitro and in vivo. Activation of the CaSR with positive allosteric CaSR modulators (calcimimetics) enhanced several inflammatory pathways in CaSR-transfected colon cancer cells, while treatment with negative allosteric CaSR modulators (calcilytics) prevented this upregulation [13,14]. The prostaglandin E_2_ (PGE_2_) pathway is one of the mediators of CaSR-induced intestinal inflammation [15]. In vivo, calcilytics reduced, while calcimimetics enhanced, specific signs and symptoms of chemically induced colitis [16]. The contradictory results of both pro- and anti-inflammatory actions of the CaSR in the colon, depending on the study, are probably due to the confounding and likely opposing effects of the CaSR in various cell types in the gut, e.g., in immune cells and epithelial cells.

The DSS (dextran sulfate sodium)-induced colitis model is the most widely used colitis model to study the pathogenesis and potential therapies of IBD, due to its simplicity and great similarity to human colitis. Several models of colitis exist, such as acute, chronic, and relapsing models, depending on the concentration of DSS, method of administration, and frequency of administration [17].

In our previous study, we used a model of rather severe colitis with 3.5% DSS to investigate pharmacological targeting of the CaSR in colitis as a preventative/therapeutic measure against this disease [16]. We showed that the calcilytic NPS 2143 reduced the clinical symptom score of mice with DSS-induced colitis, suggesting that CaSR inhibition may be a new therapeutic approach for the treatment of IBD to alleviate colitis symptoms [16]. However, the overall anti-inflammatory effect of CaSR inhibition appeared to be moderate in that model. We hypothesized that the severe colitis induced by 3.5% DSS therefore somewhat “masked” the anti-inflammatory effect of the calcilytic. Therefore, in the present follow-up study we aimed to test the hypothesis of whether pharmacological inhibition of the CaSR would have a stronger effect in a medium-grade colitis induced by a 2.5% DSS treatment.

## 2. Materials and Methods

### 2.1. Experimental Animals

All animal experiments were approved by the Ethics Committee of the Medical University of Vienna and the Austrian Federal Ministry of Education, Science and Research (GZ 66.009/0401-WF/V/3b/2017 and BMBWF-66.009/0245-V/3b/201) and carried out in accordance with the European Union Regulation on Care and Use of Laboratory Animals. We used 8-week-old female BALB/c mice (Charles River Laboratories, Inc., Sulzfeld/Grabfeld, Germany). The mice were housed in a 12 h–12 h light–dark cycle and had access to the standard semi-synthetic diet AIN-93M (LASQCdiet^®^ Rod16-A LasVendi, Soest, Germany), which contained 0.5% calcium and 13% protein, and drinking water ad libitum. One experimental unit corresponds to a single animal. Animal contamination was controlled via sentinel mice in the animal room. Treatment order was by cage but randomized every day of the treatment. Other confounders were not controlled. Except for the pathologist (see below), other group members were aware of the group allocations of the mice.

### 2.2. Experimental Design, Induction of Colitis, and Organ Collection

**Figure 1:**
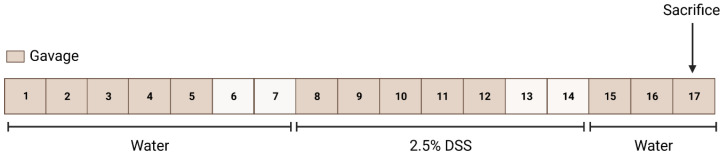
Treatment protocol. Female BALB/c mice received on weekdays a daily dose of either the vehicle (20% cyclodextrin) or the negative allosteric modulator of CaSR, NPS 2143 (10 mg/kg), via gavage (indicated by dark cells). On day 8, colitis was induced by 2.5% dextran sulphate sodium (DSS) in drinking water (ad lib.) for 7 days. After a 3-day resolution phase, the mice were sacrificed.

To induce acute colitis in the mice, we used the gold-standard model for chemically induced colitis by adding 2.5% DSS (36–50 kDa) to their drinking water for 7 days, followed by a 3-day resolution phase. The mice were randomly separated into the NPS 2143 (10 mice) or vehicle (10 mice) group. The sample size was based on a priori power calculations based on previous clinical score results. General animal welfare was the only criterion for exclusion from the study before commencement of the DSS treatment. One animal in the NPS2143 group had to be culled during the experiment for welfare reasons and was thus not included in the analysis. In the NPS 2143 group, the calcilytic NPS-2143 was administrated to the mice daily by oral gavage starting one week prior to the DSS treatment at a concentration of 10 mg/kg dissolved in 20% (2-hydroxypropyl)-β-cyclodextrin (*v*/*v*) in water (Sigma Aldrich, Darmstadt, Germany). The mice in the vehicle group were equivalently treated with 20% (2-hydroxypropyl)-β-cyclodextrin (*v*/*v*) in water via oral gavage. After the resolution phase, the mice were euthanized and colons and plasma were collected as in our previous study [16]. The experimental protocol is shown in Figure 1. Following dissection, weighing, and measuring of the cleaned colon (flushing with ice-cold PBS), small segments (~5 mm) of the proximal and distal part of the colon were collected and stored in RNALater^®^ (Invitrogen, Waltham, MA, USA) at −80 °C for gene expression studies. The rest of the colons were rolled into Swiss rolls, formalin-fixed, paraffin-embedded, and cut in 4 µm sections for histological analysis. Plasma was collected in Li-heparin-coated Microvette^®^ tubes (Sarstedt, Nümbrecht, Germany).

### 2.3. Clinical Assessment of Colitis

Clinical assessment of colitis was performed by daily weighing and monitoring of the mice during the whole experiment. The clinical score was assessed based on physical appearance, body weight loss, behavior, and feces, as described previously [16]. In brief, the clinical score was as follows: physical appearance: 0 = normal; 1 = general lack of grooming; 2 = staring coat, ocular and nasal discharges; 4 = piloerection, hunched up. Body weight loss: 0 = normal; 1 = 5–10%; 2 = 10–15%; 4 = >15%. Behavior: 0 = normal; 1 = mild depression or exaggerated response; 2 = less mobile and alert, isolated; 4 = vocalization, self-mutilation, restless, violence, inactive, cold. Feces: 0 = normal; 1 = soft, positive fecal occult blood test, Haemoccult (Beckman Coulter); 2 = very soft with visible traces of blood; 4 = visible rectal bleeding. If no stool could be collected on one day, the average score of the adjacent former and latter days was used. The clinical score was also used to define the humane endpoints for early termination of a mouse following the beginning of the DSS treatment phase. No animal reached this human endpoint in the course of the study.

### 2.4. Histology Score

The 4 µm colon sections were stained using hematoxylin-eosin according to standard protocols. After the staining, whole-section images were acquired using TissueFAXS 7 Hard- and Software (TissueGnostics GmbH, Wien, Austria), using a 20× Objective (Neo-Fluar NA 0.5; Zeiss, Oberkochen, Germany). An experienced pathologist graded the samples under blinded conditions based on the semi-quantitative evaluation of inflammation, ulceration, and mucosal remodeling, from 0–3, ranging from no abnormal observation to highest severity [16].

### 2.5. Mucin Quantification

Mucin in the colon sections was stained with Alcian blue/nuclear fast red (Vector Laboratories, Newark, CA, USA). The images of the stained sections were acquired using TissueFAXS 7 Hard- and Software, using a 20× Objective (Neo-Fluar Na 0.5). Whole-section images were then analyzed using Image J 1.53k, to determine the amount of mucin per epithelium, as described previously [16,18].

### 2.6. Cytokine Multiplex Assay

The 36-Plex Mouse ProcartaPlex Panel 1A (Thermo Fisher Scientific, Waltham, MA, USA) was used to determine chemokine and cytokine concentrations in the plasma of the mice. The targets analyzed in the experiment were GM-CSF, IFN gamma, IL-1 beta, IL-2, IL-4, IL-5, IL-6, IL-12p70, IL-13, IL-18, TNF alpha, IL-9, IL-10, IL-17A (CTLA-8), IL-22, IL-23, IL-27, G-CSF (CSF-3), IFN alpha, IL-3, IL-15/IL-15R, IL-28, IL-31, IL-1 alpha, LIF, ENA-78 (CXCL5), M-CSF, Eotaxin (CCL11), GRO alpha (CXCL1), IP-10 (CXCL10), MCP-1 (CCL2), MCP-3 (CCL7), MIP-1 alpha (CCL3), MIP-1 beta (CCL4), MIP-2, and RANTES (CCL5). The kit was used based on the manufacturer’s protocol. Concentrations were measured using a Bio-Plex 200 system (Bio-Rad, Hercules, CA, USA).

### 2.7. Immunofluorescence Staining

Ki67 staining was performed using an anti-Ki67 eFluor 570, e-Bioscience^TM^ (#41-5698-80, Thermo Fisher Scientific) antibody diluted 1:250 in 0.1% BSA in PBS. The antibody was applied overnight at 4 °C in the dark. The next day, the sections were washed with PBS + 0.05% Tween, and the cell nuclei were stained with DAPI (Sigma Aldrich) diluted 1:1000 in PBS and incubated for 10 min in the dark. CD20 and CD3 staining was performed using rabbit anti-mouse CD20 (Santa Cruz, CA, USA) and rabbit anti-mouse CD3 antibodies (Santa Cruz). Both antibodies were diluted 1:100 in PBS-T containing 0.2% Tween and 5% goat serum (Sigma Aldrich) and incubated overnight. The next day, the sections were washed with PBS and incubated for 1 h at room temperature with the secondary antibody (goat anti-mouse AlexaFluor 647) diluted 1:1000 in PBS containing 5% goat serum. DAPI staining was carried out in the same way as described above. After the staining, slides were washed briefly in distilled water and mounted with Fluoromount G (SouthernBiotech, Homewood, AL, USA) and sealed with a cover slip. Images of the whole sections were acquired with the automated Zeiss Axiolmager Z1 epifluorescence microscope equipped with TissueFAXS hard and software, using a 20× Objective (Neo-Fluar NA 0.5).

### 2.8. Quantification of Immunofluorescence

Ki67, CD3, and CD20 positive and negative cells were analyzed using TissueQuest 7 cytometry software (TissueGnostics GmbH), as described previously [16]. Individual cells were detected based on segmentation of DAPI-stained nuclei. Thresholds for positive cells were set manually and applied to all tissue sections. The analysis was performed separately for the extra-lymphatic tissue and lymph nodes.

### 2.9. Gene Expression

Total RNA was isolated from both the proximal and distal colon using the Promega ReliaPrep RNAMiniprep System (Promega, Madison, WI, USA) according to the manufacturer’s protocol. cDNA was synthesized with the High Capacity cDNA Reverse Transcription Kit (Thermo Fisher Scientific) according to the manufacturer’s protocol. Gene expression was determined using the Power SYBR Green PCR Master Mix (Thermo Fisher Scientific) on a QuantStuido 5 Real Time PCR system (Thermo Fischer Scientific). Primer sequences are provided in Supplementary Table S1. Gene expression was then calculated with the 2^−∆∆CT^ method using the average of βactin and EEF1b2 housekeeping genes and commercially available total mouse colon RNA as a calibrator (Takara, Kyoto, Japan) [19].

### 2.10. Statistical Analysis

Statistical analysis and graphing were performed using GraphPad Prism version 9.5 (GraphPad, San Diego, CA, USA), except when stated otherwise. In general, parametric tests were chosen for analysis based on the normal distribution of the data. If data were not normally distributed or if the outcome variables were discreet numbers, a non-parametric test was selected. The specific tests used for analysis are indicated in each respective figure legend.

## 3. Results

### 3.1. Treatment with NPS 2143 Had No Effect on the Clinical Score but Prevented Colon Shortening

**Figure 2:**
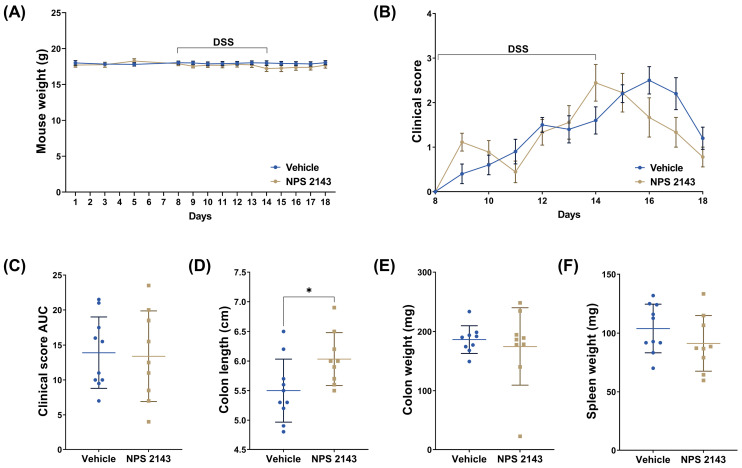
Effects of the negative allosteric modulator of CaSR NPS 2143 on macroscopic parameters of mice with DSS-induced colitis. (**A**) Body weight of the mice during the experiment. The 2.5% DSS treatment (day 8–14) is indicated in the graph. (**B**) Clinical score of the mice beginning with day 8 (start of the DSS treatment). (**C**) Cumulative clinical score over the duration of the experiment (area under the curve). (**D**) Effect of NPS 2143 on colon length, (**E**) colon weight, and (**F**) spleen weight, measured on the day of sacrifice Data are presented as mean ± SD, N = 9 (NPS 2143) and 10 (vehicle), two-sided *t*-test or Mann–Whitney U test for (**C**), * *p* < 0.05.

The weight of the mice did not change significantly over the course of the experiment and was not affected by DSS treatment for both the vehicle and NPS 2143-treated groups. The DSS-induced increase in the clinical score was not affected by either the vehicle or NPS 2143, although NPS 2143 may have slightly accelerated the resolution phase. However, the colons of the mice treated with NPS 2143 were significantly longer compared with vehicle controls, indicating a reduced overall inflammatory burden in the colons of these mice. No significant differences between mice treated with the vehicle or NPS 2143 were found in the cumulative clinical score, colon weight, or spleen weight (Figure 2C).

### 3.2. NPS 2143 Reduced Histological Inflammation

**Figure 3:**
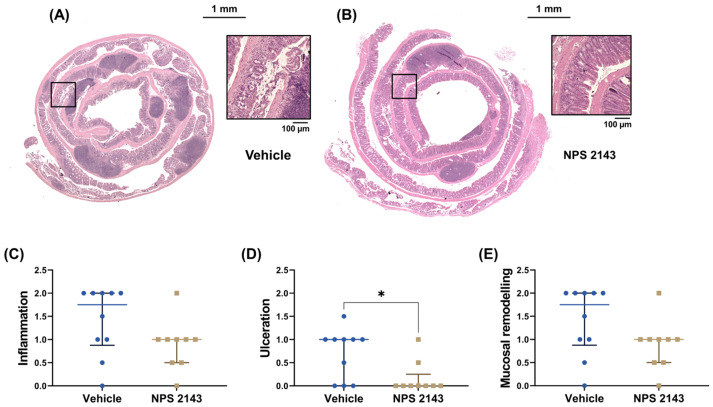
Effects of the negative allosteric CaSR modulator NPS 2143 on histological scores of colons from mice with DSS-induced colitis. Representative images of hematoxylin and eosin stainings of colon Swiss rolls from mice treated with either (**A**) vehicle or (**B**) NPS 2143. Squares in the overview indicate the region of the magnified images. The plots show (**C**) inflammation score, (**D**) ulceration score, (**E**) and mucosal remodeling of the colons from mice with DSS-induced colitis treated with either the vehicle or NPS 2143. Histological evaluation was performed by an experienced pathologist under blinded conditions. Data are presented as median ± interquartile range, N = 9 (NPS 2143) and 10 (vehicle), Mann–Whitney U test, * *p* < 0.05.

**Figure 4:**
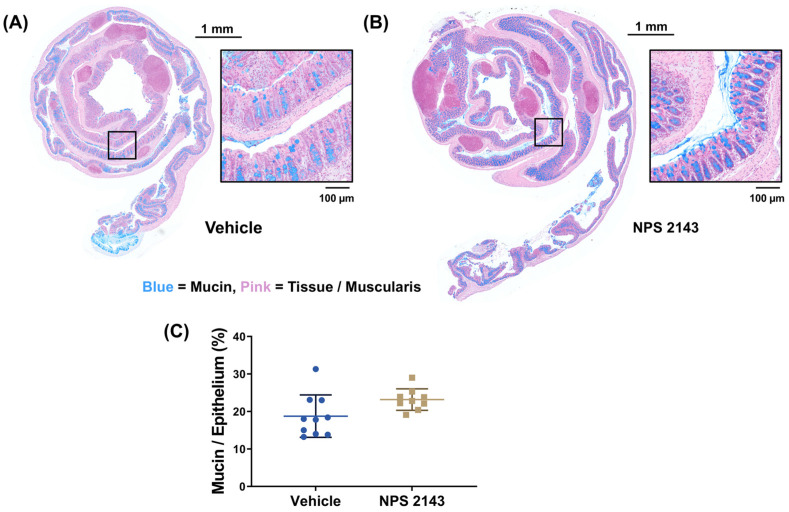
Effect of the negative allosteric CaSR modulator NPS 2143 on mucin abundance in the colonic epithelium from mice with DSS-induced colitis. Representative images of Alcian blue staining for mucin of colon Swiss rolls from mice treated with either (**A**) vehicle or (**B**) NPS 2143. Squares in the overview indicate the region of the magnified images. Contrast and brightness of Swiss rolls and fields of view (FOVs) were enhanced for the purpose of presentation only. (**C**) Quantification of mucin per epithelium of the colons from mice with DSS-induced colitis treated with either the vehicle or NPS 2143. Data are presented as mean ± SD, N = 9 (NPS 2143) and 10 (vehicle), two-sided unpaired *t*-test.

Histopathological assessment of the colons showed that treatment with NPS 2143 compared to the vehicle control significantly prevented ulceration caused by DSS (Figure 3D). Inflammation score and mucosal remodeling appeared also to be reduced by the NPS 2143 treatment, but this difference did not reach statistical significance (Figure 3). Similarly, mucin content also appeared to be higher in colons of the mice treated with NPS 2143 compared with vehicle-treated animals, though this also did not reach statistical significance (Figure 4).

### 3.3. NPS 2143 Reduced Cell Proliferation in Colonic Lymph Nodes

**Figure 5:**
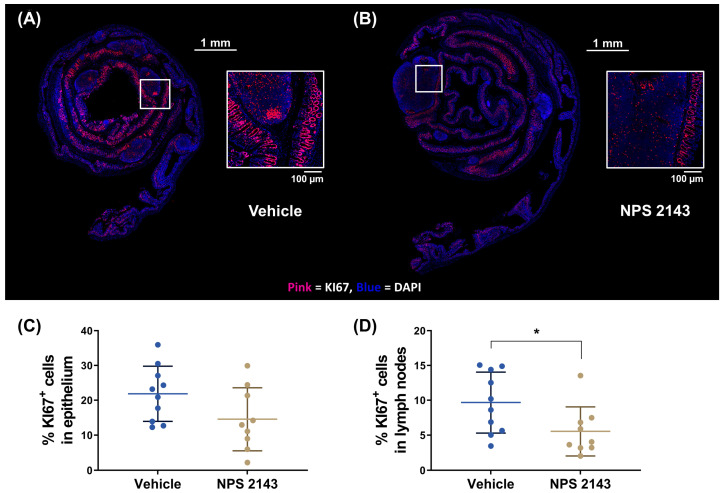
Effect of the negative allosteric CaSR modulator NPS 2143 on cell proliferation in the colons from mice with DSS-induced colitis. Representative immunofluorescence images of colon Swiss rolls from mice treated with either (**A**) vehicle or (**B**) NPS 2143 stained for KI67 (red) and counterstained with DAPI (blue). Contrast and brightness of Swiss rolls and FOVs were enhanced for the purpose of presentation only. Squares in the overview indicate the region of the magnified images. Quantification of (**C**) KI67+ cells in the tissue (**D**) and KI67+ cells in the lymph nodes of the colons from mice with DSS-induced colitis treated with either the vehicle or NPS 2143. Data are presented as mean ± SD, N = 9 (NPS 2143) and 10 (vehicle), two-sided unpaired *t*-test, * *p* < 0.05.

The abundance of proliferating cells, as determined by Ki67 staining, was analyzed separately in the epithelium and in the lymph nodes of the colon. The percentage of Ki67-positive cells was significantly lower in the colonic lymph nodes of mice treated with NPS 2143 compared with the vehicle group (Figure 5D). In the epithelium, we observed a similar reduction, but this was not statistically significant (Figure 5).

### 3.4. Immune Cell Infiltration into the Colon Was Unaffected by the Treatment

**Figure 6:**
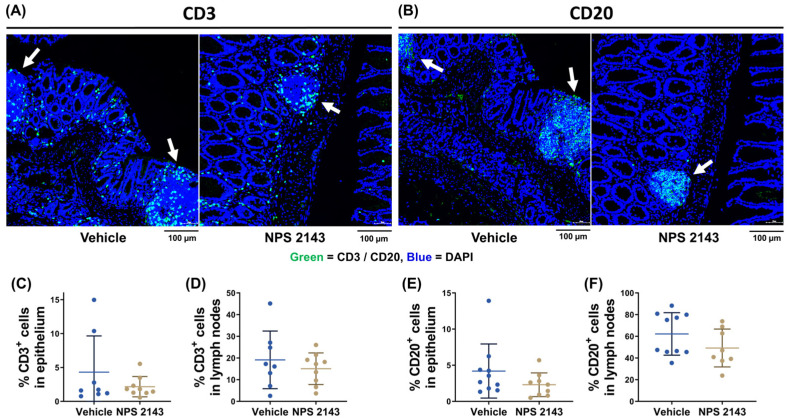
Effect of the negative allosteric CaSR modulator NPS 2143 on immune cell infiltration in the colons of mice with DSS-induced colitis. Representative immunofluorescence staining for (**A**) CD3 or (**B**) CD20 markers (green) counterstained with DAPI (blue) of colon Swiss rolls from mice treated with either the vehicle or NPS 2143. Contrast and brightness of the images were enhanced for the purpose of presentation only. Arrows indicate lymph node infiltration. Quantification of CD3+ cells (**C**) in the tissue (**D**) and in the lymph nodes of the colons from mice with DSS-induced colitis treated with either the vehicle or NPS 2143. Quantification of CD20+ cells (**E**) in the tissue (**F**) and in the lymph nodes of the colons from mice with DSS-induced colitis treated with either the vehicle or NPS 2143. Data are presented as mean ± SD, N = 9 (NPS 2143) and 10 (vehicle), two-sided unpaired *t*-test.

To assess immune cell infiltration, we counted CD3+ T cells and CD20+ B cells in the colons of the mice. Results were analyzed separately for the epithelium and the lymph nodes of the colons. NPS 2143 treatment appeared to reduce the number of T and B cells in both tissues when compared with vehicle-treated mice, but this did not reach statistical significance (Figure 6).

### 3.5. Effect of NPS 2143 Treatment on Inflammatory Markers in the Plasma and Colonic Tissue

We measured a panel of 36 cytokines and chemokines in the plasma to determine the effect of NPS 2143 on the secretion of inflammatory markers. Only 17 of the investigated markers showed detectable levels. Among these, the TNF-α level increased significantly after the treatment with NPS 2143. We did not observe any other significant changes in the concentration of cytokines or chemokines in the plasma between NPS 2143 and vehicle-treated mice (Table 1).

**Figure 7:**
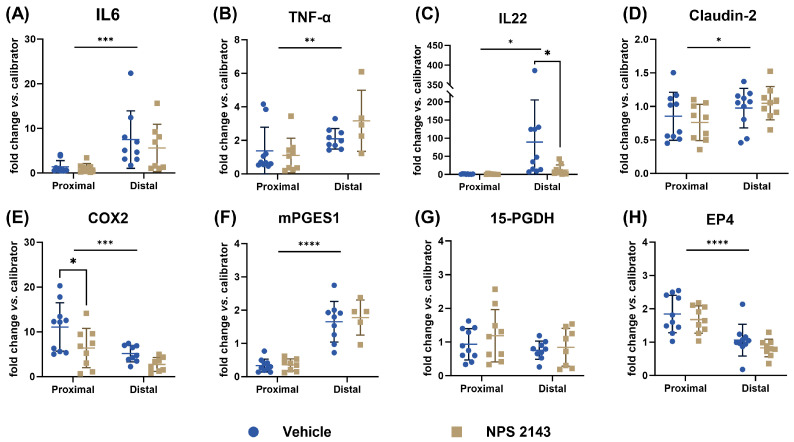
Gene expression of the inflammatory markers (**A**) IL6, (**B**) TNF-α, and (**C**) IL22, the tight-junction protein (**D**) Claudin-2, and of enzymes belonging to the PGE2 pathway, (**E**) COX2, (**F**) mPGES1, (**G**) 15-PGDH, and (**H**) EP4, in the colons of mice with DSS-induced colitis treated with either the vehicle or NPS 2143. Data are presented as mean ± SD, N = 9 (NPS 2143) and 10 (vehicle). Statistical analysis was performed with two-way ANOVA with a Šidák multiple comparison test, * *p* < 0.05, ** *p* < 0.01, *** *p* < 0.001, and **** *p* < 0.0001.

We then measured, separately for the proximal and distal part of the colon, the gene expression of the selected inflammatory markers IL6, TNF-α, and IL22, the tight-junction protein claudin-2, as well as specific members of the PGE2 pathway that were found to be affected by CaSR modulation in our previous study [15]. These were the key enzymes COX2 and PGES1, the PGE2-degrading enzyme 15-PGDH, and the EP4 receptor. For nearly all measured genes, we found significant differences in gene expression between the different sections of the colon. The expression of IL6, TNF-α, IL22, Claudin-2, and mPGES1 was significantly higher in the distal colon than in the proximal colon, while this was reversed for COX2 and EP4 expression. Treatment with NPS 2143 significantly reduced the expression of COX2 compared with vehicle in the proximal colon and the expression of IL22 in the distal colon. The expression of the other genes was not affected by NPS 2143 treatment (Figure 7).

**Figure 8:**
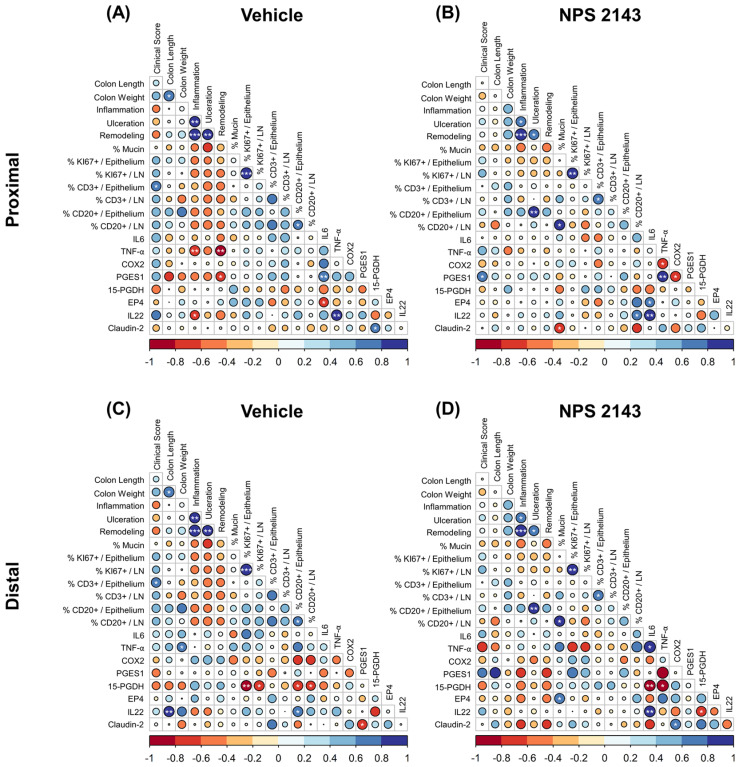
Spearman correlation matrix between relevant variables (macroscopic parameters of the whole colon and molecular markers in the proximal (**A**,**B**) and distal colon (**C**,**D**)). The color and size of the circles represents the direction and size of the correlation coefficients, respectively. Blue circles represent positive correlation (r > 0), and red circles represent negative correlation (r < 0). Small, whitish circles display insignificant correlation. Asterisks indicate significant correlation coefficients (* *p* < 0.05, ** *p* < 0.01, and *** *p* < 0.001). Statistical analysis and visualization were performed using R (version 4.2.1) and RStudio (version 2022.07.02+576) (Boston, MA, USA).

Lastly, we computed the correlations for all macroscopic parameters from the whole colon and molecular markers of the proximal and distal colon (Figure 8).

In mice treated with the vehicle only, we observed a highly significant positive correlation between the inflammation score and both the ulceration and remodeling score, indicating that these processes are closely associated. The ulceration score showed similar correlation with the remodeling score. There was also a positive correlation between the clinical score and T cell infiltration into the epithelium (Figure 8A). Mice treated with NPS 2143 showed a positive correlation between the inflammation score and both ulceration and remodeling, yet with lower correlation coefficients than in vehicle-treated mice. We also observed a strong positive correlation between the ulceration score and the CD20-positive B cell infiltration into the epithelium. Albeit not statistically significant, there was a general trend of more positive correlations between CD20-positive T cells with inflammation, ulceration, and remodeling scores, compared to vehicle-treated mice. We also observed a strong positive correlation between the mucin content and CD20-positive cells in the lymph nodes (Figure 8B).

In the proximal colon, inflammation and PGE2 pathway markers negatively correlated with TNF-α, IL22, and PGES1 when treated with the vehicle (Figure 8A). These correlations were no longer observed in the proximal colon of NPS 2143-treated mice (Figure 8B).

In the distal colon of mice treated with the vehicle only, we observed a strong negative correlation between the 15-PGDH expression and the number of KI67-positive cells and CD20-positive T cells. We also noticed a slightly positive correlation of 15-PGDH expression with inflammation, ulceration, and remodeling score (Figure 8C). Interestingly, treatment with NPS 2143 showed the exact opposite correlation, but not at a significant level. Additionally, we observed further strong negative correlations of 15-PGDH with IL6, TNF-α, and IL22 as well as a positive correlation of IL6 with TNF-α and IL22 (Figure 8D).

## 4. Discussion

Previously, we showed that inhibition of the CaSR with NPS 2143 ameliorates the clinical symptoms of mice with acute, chemically induced (3.5% DSS) severe colitis compared with vehicle-treated mice. Moreover, cinacalcet, a positive allosteric modulator of the CaSR, worsened the colitis symptoms [16]. Yet, despite a significant reduction of the clinical score in these experiments, CaSR inhibition did not have any effect on the inflammatory or histological scores of the mice. We therefore hypothesized that the protective effect of the CaSR inhibition might have been masked by the severity of the disease. Thus, in this follow-up study, we tested whether inhibition of the CaSR with NPS 2143 would be more protective in a *medium-grade* colitis induced with 2.5% DSS.

We found that treatment with NPS 2143 significantly improved histological features, prevented ulceration and, most importantly, as a clear sign of prevention of inflammation, prevented inflammation-induced shortening of the colons. Indeed, the colons of the NPS 2143-treated mice were almost as long (6.033 ± 0.447 cm) as the colons of the control mice (without DSS treatment) in our previous study (6.1 ± 0.20 cm) [16]. Colon shortening is one of the major symptoms caused by acute colon inflammation [20]. These effects were not observed in the high-grade colitis model.

Inflammatory and remodeling scores were also lower after the treatment with NPS 2143. Although the results were not statistically significant, both scores showed a lower correlation to each other, indicating a lower synergy between these processes in the presence of NPS 2143. However, in contrast to the effect seen in the high-grade colitis model, besides a shortening of the resolution phase, the effect of NPS 2143 on the clinical score was not statistically significant. This might be because the clinical score after 2.5% DSS treatment was in general much lower than the score observed in the mice treated with 3.5% DSS [16].

In another study, administration of 3% DSS in drinking water to mice significantly decreased the number of proliferating cells in their colonic epithelium [21]. The same effect was observed in a study where 5% DSS was used [22]. In our study, treatment with NPS 2143 reduced cell proliferation even further in the epithelial cells and lymph nodes; nevertheless, the result was statistically significant only in the lymph nodes. This effect was not seen in our previous study on high-grade colitis [16].

Immune cell infiltration plays a critical role in the progression of colitis [23]. During the mucosal healing process after injury in colon tissue, B cells accumulated in the damaged areas are responsible for impaired epithelial–stromal cell interactions, which are required for proper mucosal healing [24]. In IBD, T cells are dysregulated, and clearance of overactive and autoreactive cells is disrupted [25]. In the high-grade colitis model, NPS 2143 reduced immune cell infiltration into the colon. Although this immune cell infiltration was no longer observed in medium-grade colitis, correlation analysis suggested that mice treated with NPS 2143 had a stronger immune response to ulcerations, which enabled a stronger infiltration of CD20-positive cells into the epithelium, while mice with a high percentage of mucin retained CD20-positive cells in the lymph nodes.

TNF-α plays a crucial role in mediating inflammation in colitis, and it has been one of the targets for colitis treatment [26,27]. As expected by the oral treatment, NPS 2143 seems to have elicited also a systemic effect, as it led to increased levels of TNF-α in plasma but not in tissue. These results correlate with our previous findings where the TNF-α was increased in plasma but decreased in colon tissue after the treatment with NPS 2143 [16]. It has been shown that the CaSR is expressed in wide range of immune cells, such as T lymphocytes [28]. It seems that the CaSR can have pro- or anti-inflammatory effects depending on the organ or cell type it is expressed in. A study on mice with intestinal epithelial cell-specific CaSR knockout showed that lack of CaSR in the epithelium leads to diminished intestinal barrier integrity, induced stimulatory inflammatory responses, and altered composition of the gut microbiota. Additionally, mice with the CaSR knockout were more susceptible to DSS-induced inflammation, leading to colitis [11]. However, studies of our group showed that the CaSR could also have proinflammatory functions in the colon in vitro and in vivo in mice [13,14]. In the airways, the treatment of mice with NPS 2143 ameliorated the severity of allergen-induced airway hyperresponsiveness [6]. Another study showed that NPS 2143 had a protective effect against lipopolysaccharide-induced pulmonary inflammation [29]. Furthermore, the CaSR had pro-inflammatory effects in human adipose cells and tissue, where it induced the expression of inflammatory cytokines [30].

Another confounding factor that emphasizes the likelihood of a multimodal mechanism of action of CaSR inhibition on colitis is that fact that the CaSR also plays multiple physiological roles in the colon and other organs (e.g., the vasculature [31,32] and the immune system [28]), which are also (in-)directly linked to colitis. For example, the CaSR modulates a variety of ion channels in the gastrointestinal system, including TRPV6 [33] and CFTR [34,35]. Activating the CaSR reduces intestinal fluid secretion by inhibiting CFTR-mediated Cl and fluid loss. Inhibition of the CaSR could thus increase fluid secretion and lead to a faster clearance of the DSS from the colon, thus limiting disease severity. However, increased excessive fluid loss would likely increase the mortality and weight loss of the mice, which we did not observe. Still, it is likely that the other functions of the CaSR in the gastro-intestinal system, and beyond, which may have been modulated by the calcilytic, may have contributed to (or confounded) the anti-inflammatory action of CaSR inhibition.

This complication—systemic vs. local effects—makes interpretation of the exact effect of the CaSR in one specific tissue through pharmacological intervention exceedingly difficult.

Nevertheless, one of the most striking findings of our study was a strong, highly significant decrease in the gene expression of COX2 in the proximal colon following NPS 2143 treatment compared to the untreated control. This confirms our previous findings in colon cancer cells [15] and strongly suggests a direct anti-inflammatory action of CaSR inhibition in the colon. COX2 is one of the key inflammatory mediators in the intestines and is frequently upregulated in patients with colitis [36]. Interestingly, in the mice with high-grade colitis, NPS 2143 had no effect on COX2 expression but instead increased the expression of the PGE2-degrading enzyme 15-hydroxyprostaglandin dehydrogenase (15-PGDH), which is a physiological antagonist of COX2 [15,37]. In the current study, however, we did not observe any significant effects on the 15-PGDH expression after the treatment with NPS 2143. Taken together, this and our previous study [15] suggest that NPS 2143 may exert its anti-inflammatory effects via two distinct mechanisms based on the severity of colitis: in medium-grade colitis, by decreasing COX2 expression, and in high-grade colitis, by enhancing the degradation of PGE2 through increased 15-PGDH expression. However, the fact that the calcilytic so strongly downregulated COX2 expression indicates that the PGE2 pathway is central to the anti-inflammatory effect of CaSR inhibition and that the CaSR drives inflammatory processes, at least in part, by regulating PGE2 production.

Correlation analysis also revealed a strong negative correlation between 15-PGDH expression and cell proliferation in the distal colons of vehicle-treated mice, indicating that lower levels of 15-PGDH expression are associated with higher proliferation due to elevated PGE2 levels [38]. Surprisingly, in NPS 2143-treated mice, this correlation shifted from negative to positive, despite having similar 15-PGDH mRNA expression levels as in vehicle-treated mice. The exact mechanism behind this needs to be studied.

Another significant effect of the treatment with NPS 2143 was the reduction of IL22 expression in the distal colons. IL22 is a cytokine with dual effects: in acute events, it has a protective effect in the colon, promoting tissue regeneration, whereas long-term and uncontrolled activity promotes tumorigenesis [39,40]. Colitis is one of the biggest risk factors for developing colorectal cancer [41]. A decrease in IL22 following NPS 2143 treatment (together with the reduction in COX2 expression) could thus indicate that CaSR inhibition may indirectly be protective against colorectal cancer development.

We found no further significant changes after the treatment with NPS 2143 on gene expression of the other selected inflammatory markers, the tight junction protein claudin-2, and PGE2 pathway-related genes. Claudin-2 promotes colitis-associated mucosal healing, which protects against colitis-associated colon cancer [42]. However, in the histological analysis, changes were observed after NPS 2143 treatment, including mucosal remodeling. This could be explained by the fact that we measured gene expression only at the end of the experiment, after a resolution phase. It has been already shown, based on cytokine interaction and Wnt pathway genes, that the gene expression level depends on the stage of colon inflammation [43]; this may explain the lack of effect at the mRNA level despite the observed changes at the histological level.

As is common in mouse models of colitis [44,45,46], the mice were started on the putative anti-inflammatory treatment (NPS 2143 in our study) one week before the induction of the disease. Colitis is a disease characterized by periods of symptom flare-ups followed by periods of remission without symptoms. In our study, we also administered the drug before the DSS treatment, as our hypothesis is that treating patients in the remission period with NPS2143 could prevent the flare-up periods. Considering the short nature of the experiment (only two weeks total duration) as compared to the long-term effects of IBD in humans, this is a necessary compromise to ensure adequate, consistent drug levels in mice. At the same time, in IBD, CaSR antagonists may be used as a preventative adjuvant measure rather than a direct therapy, potentially preventing exacerbations. This, of course, remains to be explored in much more detail in further studies.

## 5. Conclusions

In this study, we have shown that CaSR inhibition in medium-grade colitis consistently and significantly reduced several signs and symptoms, including colon shortening, of acute inflammation. Nevertheless, the effect on the clinical symptoms of colitis was less pronounced than in mice with severe colitis. Therefore, a major finding of our study is—contrary to our initial hypothesis that CaSR inhibition might be more effective in medium-grade colitis—that the effectiveness of CaSR inhibition as an anti-inflammatory measure seems to be proportional to disease severity. Furthermore, the PGE2 pathway, as demonstrated by the strong effect of CaSR inhibition on COX2 expression, was confirmed as a pivotal pathway modulated by the CaSR. These findings, together with our previous in vivo [16] and in vitro [13,14,15] studies, provide further evidence to support the modulatory role of the CaSR in colitis and suggest its potential mechanism of action.

Given that many clinically tested calcilytics—which did not progress further in their development due to a lack of efficacy for their original indication (osteoporosis)—are available, repurposing of these drugs as potential (adjuvant) therapeutics or chemopreventatives in inflammation, including colitis/IBD, is an enticing thought. The fact that inhibition of the CaSR seems to provide a greater benefit in a setting of more severe colitis is especially interesting, as this suggests a truly disease-specific effect, which would be a further boon for a therapeutic use of these drugs.

There is still an unmet need to understand the appropriate calcilytic dose and administration route for effective colitis treatment and management. Before this can happen though, more targeted studies are needed to understand the role of CaSR in intestinal inflammation, especially as the in vivo situation is confounded by the presence of the CaSR in a variety of inflammation-associated tissue types, which would all be affected by the systemic treatment.

## Figures and Tables

**Table 1:** The effect of NPS-2143 on the concentration of different cytokines and chemokines. Two-sided unpaired *t* test. ** *p* < 0.01.

	Vehicle				NPS 2143				
Target	Mean Concentration (pg/mL)	±	SD	N	Mean Concentration (pg/mL)	±	SD	N	*p*
ENA-78 (LIX)	38.46	±	27.47	8	42.34	±	42.31	8	ns
Eotaxin	931.10	±	246.55	10	1070.00	±	142.45	10	ns
G-CSF	23.72	±	21.33	9	103.90	±	134.11	8	ns
Gro-α	15.77	±	9.56	8	22.57	±	24.95	10	ns
II-22	35.80	±	28.87	8	41.03	±	29.66	6	ns
IL-13	5.80	±	2.36	8	5.20	±	2.53	7	ns
IL-18	132.4	±	72.05	5	148.3	±	165.39	2	ns
IL-27	9.94	±	11.00	9	14.03	±	13.24	9	ns
IL-5	4.11	±	4.19	9	3.87	±	4.77	5	ns
IL-6	4.75	±	2.21	4	6.11	±	5.44	7	ns
IL-9	64.74	±	37.06	5	71.85	±	42.35	6	ns
IP-10	3.68	±	2.95	10	2.30	±	0.55	10	ns
MCP-3	69.72	±	36.49	10	59.82	±	13.55	10	ns
MIP-1α	0.88	±	0.77	9	0.95	±	0.70	8	ns
MIP-1β	1.60	±	0.75	8	1.14	±	0.37	6	ns
RANTES	22.78	±	7.52	10	19.87	±	6.03	10	ns
TNF-α	2.26	±	0.34	3	4.21	±	0.61	4	**

## Data Availability

The original contributions presented in this study are included in the article/supplementary material. Further inquiries can be directed to the corresponding author.

## Supplementary Materials

The following supporting information can be downloaded at: https://www.mdpi.com/article/10.3390/nu16244362/s1, Table S1. RT-qPCR primer list. Figure S1. Spearman correlation matrix between relevant variables (macroscopic parameters and molecular markers in the proximal colon).
