# Supplementary material for: Targeting the Calcium-Sensing Receptor in Chemically Induced Medium-Grade Colitis in Female BALB/C Mice
Source: Nutrients. 2024 Dec 18;16(24):4362. doi: 10.3390/nu16244362 (PMC11679268; doi:10.3390/nu16244362)
Supplement: Supplementary file 1 [file nutrients-16-04362-s001.zip › nutrients-3345240-supplementary.pdf]

Table S1. RT-qPCR primer list. F, Forward; R, Reverse.

Gene name		Sequence (5'→3')
**Mouse primers**		
β-actin	F:	TGACGGGGTCACCCACACTGTGCCCATCTA
	R:	CTAGAAGCATTTGCGGTGGACGATGGAGGG
Eef1β2	F:	TACATTGAGGGGTACGTGCCAT
	R:	GGTGGACCAGAAACTGCTTCA
IL6	F:	CCAGAGTCCTTCAGAGAGATACA
	R:	GCATTGGAAATTGGGGTAGGA
TNF-α	F:	TCAGCCTCTTCTATTCCTG
	R:	CAGGCTTGTCACTCGAATTT
IL22	F:	GTGCTCAACTTCACCCTGGA
	R:	TGGATGTTCTGGTCGTCACC
Claudin-2	F:	GGCTGTTAGGCACATCCAT
	R:	TGGCACCAACATAGGAACTC
COX2	F:	TGAGCAACTATTCCAAACCAGC
	R:	GCACGTAGTCTTCGATCACTATC
mPGES1	F:	AGCACACTGCTGGTCATCAA
	R:	CTCCACATCTGGGTCACTCC
15-PGDH	F:	GTTCGTCCAGTGTGATGTGG
	R:	CCTTCACCTCCGTTTTGCTT
EP3	F:	CCG GAG CAC TCT GCT GAA G
	R:	CCC CAC TAA GTC GGT GAG C
EP4	F:	ACCATTCCTAGATCGAACCGT
	R:	CACCACCCCGAAGATGAACAT

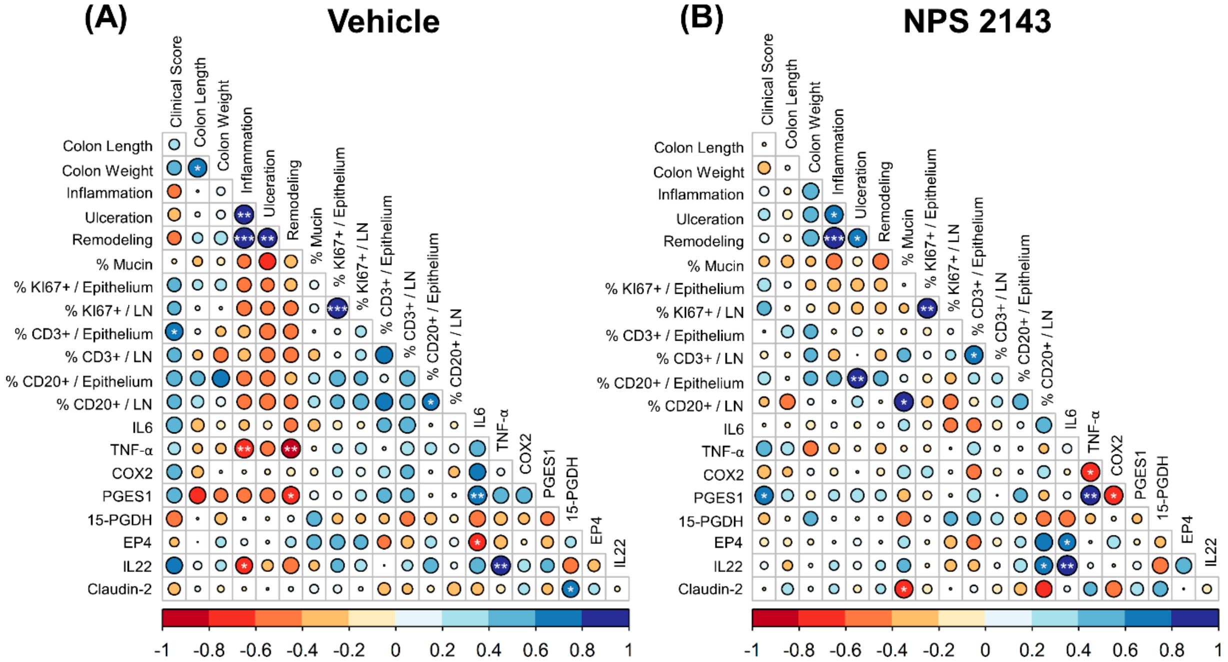

Figure S1. Spearman correlation matrix between relevant variables (macroscopic parameters and molecular markers in the proximal colon). The color and size of the circles represents the direction and size of the correlation coefficients, respectively. Blue circles represent positive correlation (r > 0), red circles represent negative correlation (r < 0). Small, whitish circles display insignificant correlation. Asterisks indicate significant correlation coefficients (* p < 0.05, ** p < 0.01, *** p < 0.001). Statistical analysis and visualization was performed using RStudio 2022.07.2 (Boston, MA, United States).
